# Integrating soil and crop metrics with precision agriculture: Pusa N Doctor app for sustainable nitrogen management in maize

**DOI:** 10.1371/journal.pone.0318678

**Published:** 2025-04-02

**Authors:** Sayantika Sarkar, Pravin Kumar Upadhyay, Abir Dey, Utpal Ekka, Sanjay Singh Rathore, Kapila Shekhawat, Md. Yeasin, Rajiv Kumar Singh, Subhash Babu, Anchal Dass, Tarik Mitran, Navin Kumar Sharma, Atul Kumar, Satendra Singh, Vinod Kumar Singh

**Affiliations:** 1 ICAR-Indian Agricultural Research Institute, New Delhi, India; 2 ICAR-Indian Agricultural Statistics Research Institute, New Delhi, India; 3 Soils & Land Resources Assessment Division, National Remote Sensing Centre, Balanagar, Hyderabad, India; 4 Krishi Vigyan Kendra, Kaushambi, India; 5 ICAR-Central Research Institute for Dryland Agriculture, Hyderabad, India; ICAR National Bureau of Soil Survey & Land Use Planning, INDIA

## Abstract

Efficient nitrogen (N) management is critical for sustaining high maize yields while minimizing environmental impacts, as conventional practices often lead to N losses, greenhouse gas emissions, and reduced eco-efficiency. To address these challenges, the “Pusa N Doctor” app was developed using dark green colour index (DGCI) for precision N management in maize. The app was further validated in experiment conducted with three N rates- 0 kg/ha (N_0_PK), 50 kg/ha (N_0_PK), and 75 kg/ha (N_75_PK) as basal, along with two splits of N at 35 and 45 DAS as per app (N_50_PK+App and N_75_PK+App) and GS^TM^ (N_50_PK + GS^TM^ and N_75_PK+GS^TM^). The plant height, leaf area index, and plant N concentration was highest in N_75_PK+App. The highest crop growth rate between 0-30 DAS was observed in the N_75_PK+App treatment (9.97 g/m²/day). Conversely, the maximum relative growth rate between 30-60 DAS was in the N_50_PK+App, while the lowest was in N_75_PK+App. The highest harvest index of 35.13% was in N_50_PK+App. Except for N_75_PK+App and recommended dose of fertilizer (RDF), the partial N balance was close to 1, with a minimum value of 0.87 in N_75_PK+App. The lowest virtual N was in N_50_PK+App (0.45), while in N_75_PK+App it was 2.16 times higher than RDF. All N fertilized treatments except N_50_PK+App witnessed increased cost of cultivation over RDF. N_50_PK+App had 29.5% lower GHGI of N_2_O, with 11.6% and 13.3% higher energy and GHG-based eco-efficiency respectively than RDF. Thus, applying 50 kg N as basal along with its 2 splitting as per Pusa N Doctor, optimizes maize-growth, N use efficiency, eco-efficiency, and reduces GHG emissions.

## 1. Introduction

Despite notable advancements in agricultural practices, the challenge of imbalanced fertilizer application continues to persist in Indian agriculture, particularly due to a pronounced reliance on nitrogen (N) fertilizers [1]. Farmers, often driven by the perceived benefits of increased yields, tend to apply nitrogen disproportionately, neglecting the balanced use of other essential nutrients. This skewed fertilization practice not only reduces nutrient use efficiency but also imposes a substantial financial burden on farmers, as excessive nitrogen application often results in diminishing economic returns [2,3]. Moreover, the environmental repercussions of such practices are profound. Over-application of nitrogen contributes to nitrate leaching into groundwater, posing risks to water quality and public health. Elevated nitrate levels in drinking water are linked to severe health issues, including methemoglobinemia in infants [4,5]. Additionally, excessive nitrogen disrupts soil health by altering its chemical and biological properties, leading to long-term productivity decline [6]. These environmental consequences, coupled with reduced nutrient use efficiency, amplify the challenges faced by Indian agriculture. In the broader context, the cumulative impact of these issues undermines agricultural productivity, threatening the sustainability of food production systems and posing a significant risk to global food security [7].

Therefore, precision nutrient management (PNM), adhering to the 4R nutrient stewardship principles—applying the right fertilizer source, at the right rate, at the right time, and in the right place [8], is crucial for optimizing N use, directly impacting both economic returns and environmental sustainability. This approach aims to enhance crop productivity, boost farmer profitability, protect the environment, and improve both sustainability and nutrient use efficiency [9,10].

Several tools for real-time N management have been developed, such as GreenSeeker, SPAD (The Soil Plant Analysis Development) chlorophyll meter, and LCC (leaf colour chart), which predict crop N needs based on leaf reflectance and greenness [11,12]. These tools evaluate leaf N levels, which correlate with photosynthesis and biomass production, making them sensitive indicators of crop N demand throughout the growing season [13]. The GreenSeeker™ (GS^TM^) (Trimble Ltd., Sunnyvale, CA, USA) is particularly effective for making mid-season N recommendations based on the normalized difference vegetation index (NDVI). But the high cost of GS^TM^ and SPAD 502Plus meter (Konica Minolta Inc., New Jersey, USA) and the need for complex calculations limit their adoption among Indian farmers [14, 15]. In contrast, the LCC is an affordable, user-friendly tool for monitoring leaf greenness as an indicator of N status [16,17]. However, LCC’s limitations include its binary N recommendations—whether to apply or not, without specifying the dosage—and its reliance on the farmer’s subjective colour perception [18]. According to a 2018 study by US media agency Zenith, with over half of India’s population owning camera-equipped smartphones, mobile-based applications offer a promising digital platform to estimate leaf N status. Therefore, an attempt has been made to develop a smartphone app “Pusa N Doctor”, based on the dark green colour index (DGCI) [19], which can provide real-time N recommendation in maize by analyzing images of crop leaves taken with a smartphone camera. This study aims to validate the app, with respect to maize growth parameters, yield attributes, financial advantage, N use efficiency, nitrous oxide (N_2_O) emission, and eco-efficiency.

## 2. Materials and methods

### 2.1. Study site and climatic conditions

**Fig 1:**
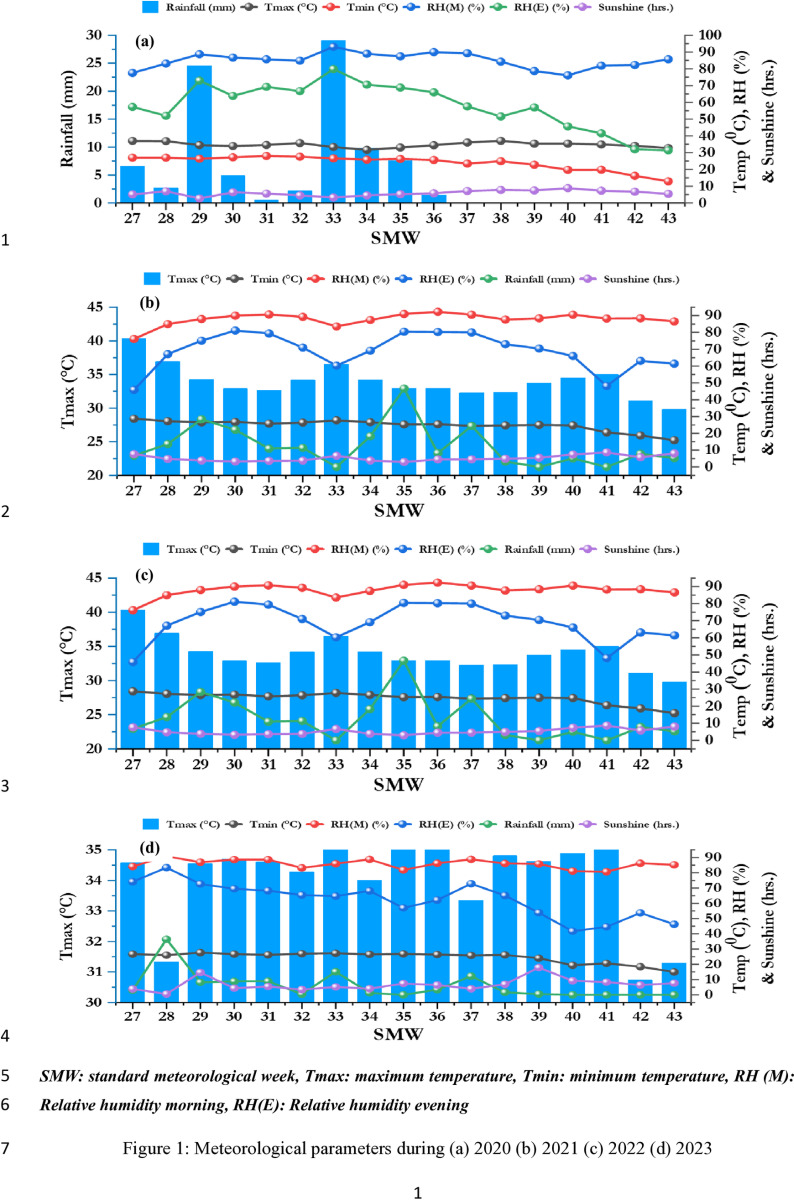
Meteorological parameters during (a) 2020; (b) 2021; (c) 2022; (d) 2023.

Field experiments were conducted with maize during 2020, 2021 and 2022 in the experimental field of ICAR- Indian Agricultural Research Institute (ICAR-IARI), New Delhi (28˚4′ N, 77˚12′ E, 228.6 m above sea level). The study area experiences a subtropical, semi-arid climate, marked by hot, dry summer and cold winter. More details regarding the weather parameter during study period is presented in Fig 1. The soil at the experimental site was sandy loam (Typic Haplustept) with a slightly alkaline pH of 7.7. The initial soil properties comprised of a mildly alkaline soil reaction (1:2.5 soil-to-water) of 8.1, an EC of 0.35 dS/m, low organic carbon content of 0.44%, low available N at 185 kg/hectare, and medium levels of available phosphorus (P) and potassium (K), measuring 12.8 kg/hectare and 205 kg/hectare, respectively. Before the initiation of the validation experiment in the *kharif* 2023, initially the top 0-15 cm soil contained 0.49% organic carbon, 223 kg/ha available N, 16.8 kg/ha available P, and 242 kg/ha available K.

### 2.2. App development

#### 2.2.1. Treatment details.

The three-season experiment for the app development during 2020, 2021, and 2022 were conducted with six popular varieties/hybrids of maize crop (PC3, PC4, AH-4271, DKC-9164, PJMH-1, PMH-1) along with seven levels of N application, i.e., 0, 40, 80, 120, 160, 200 and 240 kg/ha in a factorial randomized complete block design (RCBD) replicated five times. A basal dose of 75 kg/ha of P_2_O_5_ and K_2_O was added in all the plots irrespective of the treatments. In the treatment with 40 kg/ha N, the entire N amount was applied as a basal dose. For the 80 kg/ha N treatment, 40 kg/ha N was applied as a basal dose and an additional 40 kg/ha N at 35 days after sowing (DAS). For 120 kg/ha N and above, one-third of N was used as basal, one-third at 35 DAS, and one-third at 45 DAS.

#### 2.2.2. Smartphone app development.

**Fig 2:**
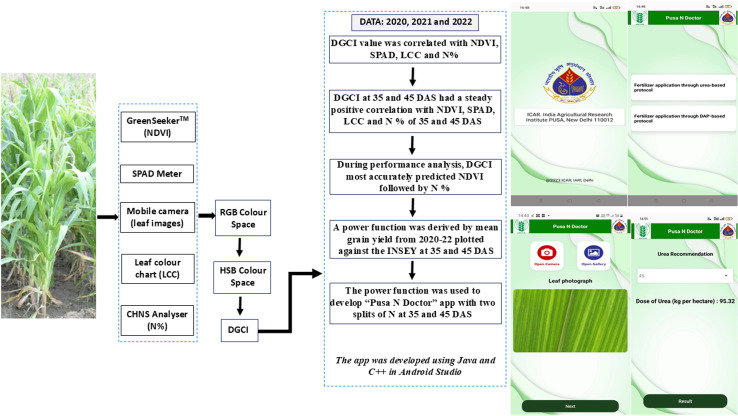
Schematic diagram showing Pusa N Doctor app implementation. Here, DGCI is the dark green colour index; RGB is red, green and blue colour space; HSB is the hue, saturation and brightness colour space; and INSEY is the in-season estimated yield.


The app development process is depicted in Fig 2. The DGCI was calculated [19] as:

$$\text{DGCI}=\left[\left(\frac{\text{H}}{60}-1\right)+\left(1-\text{S}\right)+\left(1-\text{B}\right)\right]/3$$ (1)

S = C/ max (R, G, B)

H = 60×(G-B)/C (when, max (R, G, B) = R)

H = 60×(2+(B-R)/C) (when, max (R, G, B) = G)

H = 60×(4+(R-G)/C) (when, max (R, G, B) = B)

C = max (R, G, B)-min (R, G, B)

B = max (R, G, B)

In the above equation, H: hue, S: saturation, B: brightness; C: chroma; and R, G, B denotes the normalized red, green, blue levels respectively.

The INSEY which is the in-season estimated yield was computed as:


$$\text{INSEY}=\frac{\text{DGCI }}{\text{Number of GDD}>0}$$ (2)

Where, GDD is the growing degree days.

Response Index (RI) was deduced according to the below equation:


$$\text{RI}=\frac{\text{DGCI }\left(\text{ENRICH}\right)}{\text{DGCI }\left(\text{TEST}\right)}$$ (3)

Where, DGCI (ENRICH) is the DGCI value of the N enriched strip maintained by supplying 240 kg N/hectare.

The empirically derived power function relating INSEY and grain yield at 35 and 45 DAS is represented as:


$${\text{YP}}_{0}=\text{a }\times {\left(\text{Estimated yield}\right)}^{\text{b}}$$ (4)

Where, YP_0_ is the potential yield without additional fertilizer; a, and b are constants.

The achievable yield with added N fertilizer to the test plots (YP_N_) was assessed by multiplyingYP_0_ by the RI as described below:


$${\text{YP}}_{\text{N}}={\text{YP}}_{0}\times \text{RI}$$ (5)

Finally, fertilizer N prescription was calculated as follows:


$$\text{Fertilizer N dose}=10\times 1.43\times \frac{{\text{YP}}_{\text{N}}-{\text{YP}}_{0}}{0.5}$$ (6)

Where, 1.43 represents the mean maize grain N concentration and 0.5 is the achievable efficiency factor in South-Asia [20].

In the validation experiment, Pusa N Doctor validated against GS^TM^, and for the calculation of N dose with GS^TM^, the DGCI values were simply replaced by the NDVI values in equations (2) and (3).

### 2.3. Validation experiment

#### 2.3.1. Treatment details and crop management.

The validation experiment was conducted during *kharif* 2023 in RCBD with 6 treatments and 4 replications. The treatments comprised of 0 kg/ha (N_0_PK), 50 kg/ha (N_50_PK) and 75 kg/ha (N_75_PK) of N, in addition to 75 kg of P_2_O_5_ and K_2_O incorporated as basal (Table 1). Additionally, N doses of 41.9 kg/ha and 30 kg/ha were applied in two splits at 35 and 45 DAS respectively following precision N management-based recommendations through android-based Pusa N Doctor application in N_50_PK+App while 58.6 kg/ha and 41.2 kg/ha were applied in N_75_PK+App. In the GreenSeeker™ based treatments, two splits of 39.4 kg/ha and 36.4 kg/ha of N were applied respectively at 35 and 45 DAS in N_50_PK+GS^TM^, whereas 35.6 kg/ha and 33.7 kg/ha were applied in N_75_PK+GS^TM^. These treatments were compared with the recommended dose of fertilizer (RDF), where 150 kg/ha of N was used in equal splits as basal, at 30 DAS and 50 DAS.

**Table 1:** Treatments details of the validation plot.

Treatment	Details
N_0_PK	No N, full dose of P and K at the time of sowing
N_50_PK + App	50 kg N along with full dose of P and K at the time of sowing, remaining two splits of N as per Android based prescription at 35 and 45 DAS
N_50_PK + GS™	50 kg N along with full dose of P and K at the time of sowing, remaining two splits of N as per GSTM prescription at 35 and 45 DAS
N_75_PK + App	75 kg N along with full dose of P and K at the time of sowing, remaining two splits of N as per Android based prescription at 35 and 45 DAS
N_75_PK + GS^TM^	75 kg N along with full dose of P and K at the time of sowing, remaining two splits of N as per GSTM prescription at 35 and 45 DAS
RDF	Recommended dose of fertilizer [(N in three splits: 75 kg as basal, 37.5 at knee high stage and 37.5 at Pre-tasseling stage), full dose of P and K at the time of sowing]

Initial soil properties of the validation experiment are given in Table 2. In all experimental treatments, PJMH1 maize cultivar, released jointly by ICAR-IARI, New Delhi and Jawaharlal Nehru Krishi Vishwa Vidyalaya (JNKVV), Jabalpur, was selected for cultivation. The land preparation process involved deep summer ploughing before monsoon arrival, followed by, one ploughing, one discing, one planking to ensure good tilth. To prevent surface water flow from one plot to another, bunds were built around each plot. The crop was sown using tractor drawn opener with 60 cm x 20 cm spacing, and 20 kg/ha seed rate, on 4th July, 2023. During the entire cropping season three irrigations of 5 cm depth each were given on 3^rd^ August (30 DAS), 18^th^ August (47 DAS), and 30^th^ August (57 DAS), 2023, when there was a dry spell.

**Table 2:** Initial soil properties of the experimental field.

Soil Properties	Soil depth (cm)	
	0-15 cm	15-30 cm
Bulk density (BD) (g/cm³)	1.55	1.68
Soil organic carbon (%)	0.51	0.45
Available nitrogen (kg/ha)	223	133.3
Available phosphorus (kg/ha)	16.8	10.7
Available potassium (kg/ha)	242	171
pH (1:2.5 soil: water)	8.1	8.4
Electrical conductivity (EC) (dS/m)	0.35	0.37

#### 2.3.2. Measured and calculated parameters.

2.3.2.1.Growth parameters: At 30, 60, and 90 days after sowing (DAS) and at harvest, three plants were randomly selected from each plot to measure plant height. The measurements were taken using a meter scale, and the average height was recorded in centimetres (cm). During the tasselling and harvest stages, height was measured from the ground to highest fully open leaf, while at the knee-height stage, the measurement extended from the ground to the tip of the folded leaf.

Leaf area was measured at 30 and 60 days after sowing (DAS) by removing the leaves from the same three maize plants previously selected for dry matter analysis. The leaves were then measured using a leaf area meter (Model: LI-COR-3100). This data was used to calculate the leaf area index (LAI) using the following formula:


$$\text{LAI}=\frac{\text{Leaf area per plant }\left({\text{cm}}^{2}\right)}{\text{Planting geometry }\left({\text{cm}}^{2}\right)}$$ (7)

Dry matter accumulation was monitored at 30 DAS, 60 DAS, 90 DAS and at harvest by pulling out three maize plants per plot. The plants were initially air-dried for 7 to 8 days, followed by oven drying at 65°C for two days until a stable mass was achieved, which was recorded as grams of dry matter/ plant. The crop growth rate (CGR) and relative growth rate (RGR) was then worked out by the following formula:


$$\text{CGR}=\frac{{\text{W}}_{2}-{\text{W}}_{1}}{{\text{T}}_{2}-{\text{T}}_{1}}\times \frac{1}{\text{A}}$$ (8)

The relative growth rate was calculated out with the following formula [21]:


$$\text{RGR}=\frac{{\text{l}}_{\text{n}}{\text{W}}_{2}-{\text{l}}_{\text{n}}{\text{W}}_{1}}{{\text{T}}_{2}-{\text{T}}_{1}}$$ (9)

Where,

l_n_: the natural logarithm, W_1_ and W_2_ are dry weight (g) of plants at time T_1_ and T_2_, respectively.

T_2_ – T_1_ is the interval of time in days.

A is the land area (m^2^) occupied by plants

2.3.2.2.Shelling percentage and harvest index: To determine crop yield, the harvested area excluded two border rows on each side and 0.5 m strip along the length of the plot. Once the stover was separated and the husk along with the silk were removed, cobs from every net plot were sun-dried and the weight was expressed as cob yield (t/ha). The maize stover was cut at ground level and also sun-dried before being weighed. The total weight of the harvested materials (both cobs and stover) from each net plot was measured and reported as biological yield (t/ha). Cobs from each net plot were shelled, and the grains were sun-dried. The final grain yield was then adjusted to 14.5% moisture content and expressed in tons/hectare (t/ha).

Shelling percentage was calculated as follows:


$$\text{Shelling }\left(\text{\%}\right)=\frac{Grainyield\left(t/ha\right)}{Cobyield\left(t/ha\right)}\times 100$$ (10)

The harvest index was calculated using the following equation:


$$\text{Harvest Index }\left(\text{\%}\right)=\frac{\text{Grain yield }\left(\text{t}/\text{ha}\right)}{\text{Biological yield }\left(\text{t}/\text{ha}\right)}\times 100$$ (11)

2.3.2.3.Plant analysis: After sun-drying, plant samples collected at 40 and 50 DAS, harvested maize grain and stover samples were further dried in a hot air oven at 60°C for 24 hours to reach a constant weight. The dried samples were then ground using a Retsch mixer mill MM 400 and passed through a 40-mesh sieve. Further, 0.5 g of sample from the plots were used to determine N concentration. N content (%) in maize plant at 40 and 50 DAS, grain and stover was analyzed using the modified Kjeldahl method [22] by a CHNS analyser (Carbon, Hydrogen, Nitrogen, and Sulfur analyser) (Euro EA-3000). The N uptake by maize grain (Grain N uptake), and stover (Stover N uptake) was calculated by multiplying the N content (%) in maize grain with grain yield (kg/ha). Total N uptake was finally expressed as the sum of grain N uptake and stover N uptake.

2.3.2.4.Nitrogen use efficiency: Following formulae were used for the computation of physiological efficiency of N (PE_N_) [23]:


$${\text{PE}}_{\text{N}}=\frac{{\text{Grain yield}}_{\text{N}}-{\text{Grain yield}}_{\text{C}}}{{\text{Total N uptake}}_{\text{N}}-{\text{Total N uptake}}_{\text{C}}}$$ (12)

The partial N balance (PNB_N_) was computed as per the following equation (24):


$${\text{PNB}}_{\text{N}}=\frac{{\text{Total N uptake}}_{\text{N}}}{{\text{F}}_{\text{N}}}$$ (13)

The internal utilization efficiency of N (IUE_N_) was calculated as per Parco et al. [25]; Qiu et al. [26]:


$${\text{IUE}}_{\text{N}}=\frac{{\text{Grain yield}}_{\text{N}}}{{\text{Total N uptake}}_{\text{N}}}$$ (14)

The virtual N factor (VNF) was calculated by using following formula [27]:


$$\text{VNF}=\frac{\text{N loss to the environment}}{{\text{Grain N Uptake}}_{\text{N}}}$$ (15)

Where the N loss to the environment was calculated as the difference between N input (fertilizer + soil N supply) and N output (Total plant N uptake). The total N uptake by the no N control plot was assumed as the soil N supply.

In Equations (13) and (14), Total N uptake_N_ represents the total N uptake by the maize stover and grain from the plots applied with N (kg/ ha), Total N uptake_C_ represents the total N uptake by the maize stover and grain from the control (kg/ ha), F_N_ denotes the dose of N used (kg/ ha), Grain yield_N_ denotes grain yield from the plots applied with N (kg/ha), Grain yield_C_ denotes grain yield from control plot and Grain N Uptake_N_ represents the grain N uptake from the plots applied with N (kg/ ha).

2.3.2.5.Economic analysis: The calculation of the cost of cultivation (CoC) was done based on current input prices. The total cost accounted for various inputs like fertilizers, irrigation, seeds, and agronomic activities, including soil cultivation, sowing, pest control, and harvesting. Human labour costs were computed based on an eight-hour workday, as outlined by the Indian labour laws in addition to hours/hectare of machinery time for each farm activity. Total costs were determined by summing labour, machinery time, fuel, and electricity for each activity. The rental value of the land was also factored into the cultivation costs. The cost of GS^TM^ was considered as US $ 960, with life span of 16 years, which amounts to additional US $ 60 for a single year for the GS^TM^ based treatments. Saving of cost over RDF ($/ha) was computed as the difference in CoC between RDF and the rest of the treatments. The economic assessments were carried out in Indian rupees and subsequently transformed to US $.

2.3.2.6.Greenhouse gas intensity of nitrous oxide and eco-efficiency index: Greenhouse gas (GHG) emitted by the treatments were assessed using the Cool Farm Tool (CFT v1.7.1) [28], incorporating multiple globally validated models into a unified system to estimate emissions resulting from agricultural inputs in crop production systems. The Greenhouse gas intensity (GHGI) of N_2_O was calculated as:


$${\text{GHGI of N}}_{2}\text{O }\left({\text{kg CO}}_{2}-\text{eq}/\text{t}\right)=\frac{{\text{N}}_{2}\text{O emission }\left({\text{kg CO}}_{2}-\text{eq}/\text{ha}\right)}{\text{Grain yield}\left(\text{t}/\text{ha}\right)}$$ (16)

Eco-efficiency integrates both the financial and environmental dimensions of a production system. It measures the effectiveness of a cropping system in boosting economic output while minimizing environmental impacts, such as energy consumption (equation (17)) and GHG emissions (equation (18)) from agriculture [29, 30].


$$\text{Eco}-\text{efficiency index}\left(\text{US \$}/\text{MJ}\right)=\frac{\text{Gross returns }\left(\text{US \$}/\text{ha}\right)}{\text{Total energy input }\left(\text{MJ}/\text{ha}\right)}$$ (17)

$$\text{Eco}-\text{efficiency index}\left(\text{US \$}/{\text{kg CO}}_{2}-\text{eq}\right)=\frac{\text{Gross returns }\left(\text{US \$}/\text{ha}\right)}{\text{GHG emission }\left({\text{kg CO}}_{2}-\text{eq}/\text{ha}\right)}$$ (18)

Energy input was categorized into direct (labour, fuel, electricity) and indirect (seeds, fertilizers, machinery, chemicals) energy inputs. It was further divided into renewable (seeds, labour) and non-renewable sources (fuel, electricity, machinery, fertilizers, chemicals). Energy consumption was determined by applying energy coefficients [31, 32, 33, 34] to inputs.

### 2.4. Statistical analysis

The data on various parameters were analyzed using analysis of variance (ANOVA) for RCBD. The differences between treatment means were tested at P < 0.05 according to Fisher’s LSD test [35] and to depict them boxplots were generated for harvest index, N use efficiency indices, and GHGI. Ridgeline plot was developed for the eco- efficiency index, where the black line within the plot represents the mean, the different lowercase letters within the plot correspond to the significant difference of the treatments at p <  0.05 according to LSD test, purplish colour indicates lower eco- efficiency index value, and as we move towards yellowish tinge, we can find a comparatively higher eco- efficiency index. All the statistical analysis was done in R programming (version 4.2.1) and MS-Excel (Microsoft corporation, 2019).

## 3. Results

### 3.2. Plant height and leaf area index

There was significant effect of N management practices on the plant height of maize at 60 DAS, 90 DAS and at harvest (Table 3). The maize plant height in different treatments were at par with each other at 30 DAS. The highest maize height was in N_75_PK+App treatment at 30, 60, 90 DAS and at harvest. At 60 DAS, though plant height was highest under N_75_PK+App but it has no significant difference with RDF, followed by N_75_PK+GS^TM^, N_50_PK+GS^TM^. At 90 DAS and harvest, the treatments had a comparable height, which was significantly higher than the control.

**Table 3:** Maize plant height across the treatments at 30 DAS, 60 DAS, 90 DAS and at harvest.

*Treatments	Maize plant height (cm)			
	30 DAS	60 DAS	90 DAS	Harvest
N_0_PK	71.0^a^	165.4^e^	170.1^b^	172.3^b^
N_50_PK + App	75.1^a^	191.9^d^	200.1^a^	201.6^a^
N_50_PK + GS^TM^	72.8^a^	195.3 cd	203.3^a^	204.6^a^
N_75_PK + App	78.1^a^	206.0^a^	207.5^a^	208.8^a^
N_75_PK + GS^TM^	75.8^a^	198.7^bc^	203.9^a^	205.4^a^
RDF	72.3a	203.4^ab^	207.0^a^	208.1^a^

*For treatment details, please refer Table 2. Means followed by different lowercase letter within each column are significantly different (p <  0.05) according to LSD test.

The LAI of maize was significantly influenced by the N management practices with the highest being under N_75_PK+App treatment at both 30 DAS (1.40) and 60 DAS (4.20) (Table 4). There was no significant difference in LAI under N_75_PK+App and N_75_PK+GS^TM^, but it was 18.6% higher than RDF at 30 DAS. But, at 60 DAS, N_75_PK+App and N_75_PK+GS^TM^ had a comparable LAI with RDF. Though the N_50_PK+App had a comparatively lower LAI, but it was statistically at par with N_50_PK+GS^TM^.

**Table 4:** Leaf area index (LAI) of maize across the treatments.

*Treatments	Maize leaf area index (LAI)	
	30 DAS	60 DAS
N_0_PK	0.92^c^	3.21^c^
N_50_PK + App	1.18^b^	4.01^b^
N_50_PK + GS^TM^	1.17^b^	4.01^b^
N_75_PK + App	1.40^a^	4.20^a^
N_75_PK + GS^TM^	1.39^a^	4.19^a^
RDF	1.18^b^	4.20^a^

*For treatment details, please refer Table 2. Means followed by different lowercase letter within each column are significantly different (p <  0.05) according to LSD test.

### 3.3. Crop growth rate, relative growth rate, shelling percentage and harvest index

There was a significant difference amongst the N fertilized plots with respect to the CGR and RGR of maize (Table 5). Though between 0-30 days’ time interval the highest CGR (9.97 g/m^2^/day) was in N_75_PK+App treatment, it was at par with N_75_PK+GS^TM^ (8.86 g/m^2^/day) and RDF (9.45 g/m^2^/day), which again had no significant difference with N_50_PK+App (8.50 g/m^2^/day) and N_50_PK+GS^TM^ (8.67 g/m^2^/day). On the contrary, the maize RGR between 30-60 DAS in N_50_PK+App was the maximum, while N_75_PK+App had the lowest RGR of 0.0351 g/g/day. Between 30-60 DAS and 60-90 DAS there was at par CGR in the N fertilized plots which differed significantly with the control. Similar was the result for the RGR at 60-90 DAS as well.

**Table 5:** Crop growth rate (CGR) and relative growth rate (RGR) of maize across the treatments.

*Treatments	Maize CGR (g/m^2^/day)			Maize RGR (g/g/day)	
	(0-30) DAS	(30-60) DAS	(60-90) DAS	(30-60) DAS	(60-90) DAS
N_0_PK	5.33^c^	10.78^b^	4.82^b^	0.0369^a^	0.0099^b^
N_50_PK + App	8.50^b^	17.64^a^	23.64^a^	0.0384^a^	0.0233^a^
N_50_PK + GS^TM^	8.67^b^	18.22^a^	23.67^a^	0.0378^a^	0.0233^a^
N_75_PK + App	9.97^a^	18.56^a^	24.36^a^	0.0351^a^	0.0225^a^
N_75_PK + GS^TM^	8.86^ab^	18.08^a^	23.88^a^	0.0372^a^	0.0233^a^
RDF	9.45^ab^	18.22^a^	24.13^a^	0.0352^a^	0.0236^a^

*For treatment details, please refer Table 2. Means followed by different lowercase letter within each column are significantly different (p <  0.05) according to LSD test.

**Fig 3:**
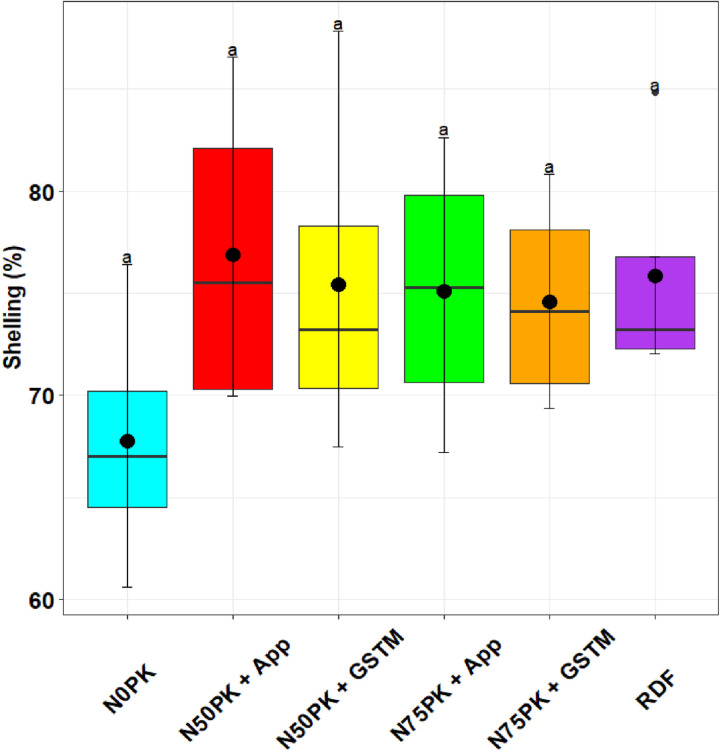
Effect of treatments on shelling (%) of maize.

**Fig 4:**
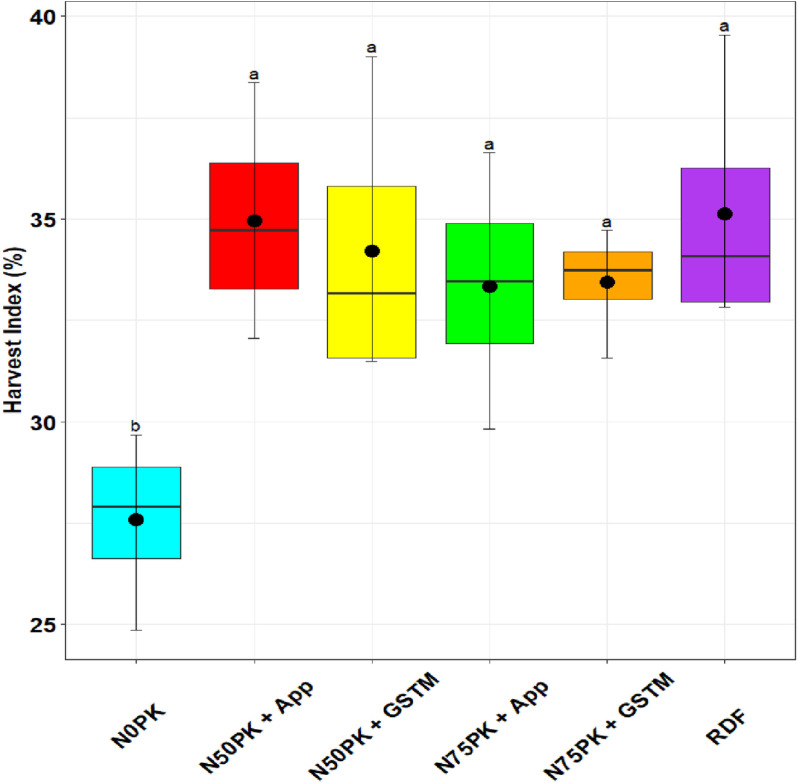
Effect of nitrogen management practices on Harvest Index (%) in maize. The different lowercase letters correspond to the treatments are significantly different at p <  0.05 according to LSD.

The shelling percent (Fig 3) in maize was highest in N_50_PK+App (76.88%), which was statistically at par with the control (67.75%) and other N fertilized plots. The lowest harvest index (Fig 4) was in the control plot (27.6%), followed by N_75_PK+App treatment (33.3%), which had no statistically significant difference with the other precision N based treatments including N_50_PK+App (35.13%) which had the highest harvest index.

### 3.4. Nitrogen content in plant at 40 and 50 DAS, and in grain and stover at harvest

There was no statistically significant difference between the treatments with respect to their plant N (%) at both 40 and 50 DAS (Table 6). The highest N content in maize plant at 40 DAS was in the plots applied with 75 kg N as basal (N_75_) N_75_PK +  App (2.79%) and N_75_PK +  GS^TM^ (2.71%). Similar was the trend for 50 DAS as well, where, N_75_ had the maximum N % of 1.69% followed by RDF (1.67%) and the plots applied with 50 kg N as basal (N_50_) (1.65%).

**Table 6:** N (%) in maize plant at 40 DAS, 50 DAS, N (%) in maize grain and stover at harvest across the treatments.

*Treatments	N (%) in maize plant at 40 DAS	N (%) in maize plant at 50 DAS	N (%) in maize grain at harvest	N (%) in maize stover at harvest
N_0_PK	2.58	1.62	1.68	0.43
N_50_PK + App	2.60	1.65	1.73	0.46
N_50_PK + GS^TM^	2.63	1.65	1.73	0.46
N_75_PK + App	2.79	1.69	1.74	0.48
N_75_PK + GS^TM^	2.71	1.69	1.74	0.47
RDF LSD (P ≤ 0.05)	2.57 NS	1.67 NS	1.73 NS	0.47 NS

*For treatment details, please refer Table 2, LSD: least significant difference, NS: statistically non-significant.

All the Precision N based treatments were at par with the RDF in their grain and stover N content (Table 6). N_75_PK +  App had the highest grain and stover N of 1.743% and 0.479% respectively, followed by N_75_PK +  GS^TM^ (grain and stover N content of 1.736% and 0.473% respectively). The grain and stover N content progressively decreased for RDF, N_50_PK +  GS^TM^, and N_50_PK +  App.

### 3.5. Nitrogen use efficiency

**Fig 5:**
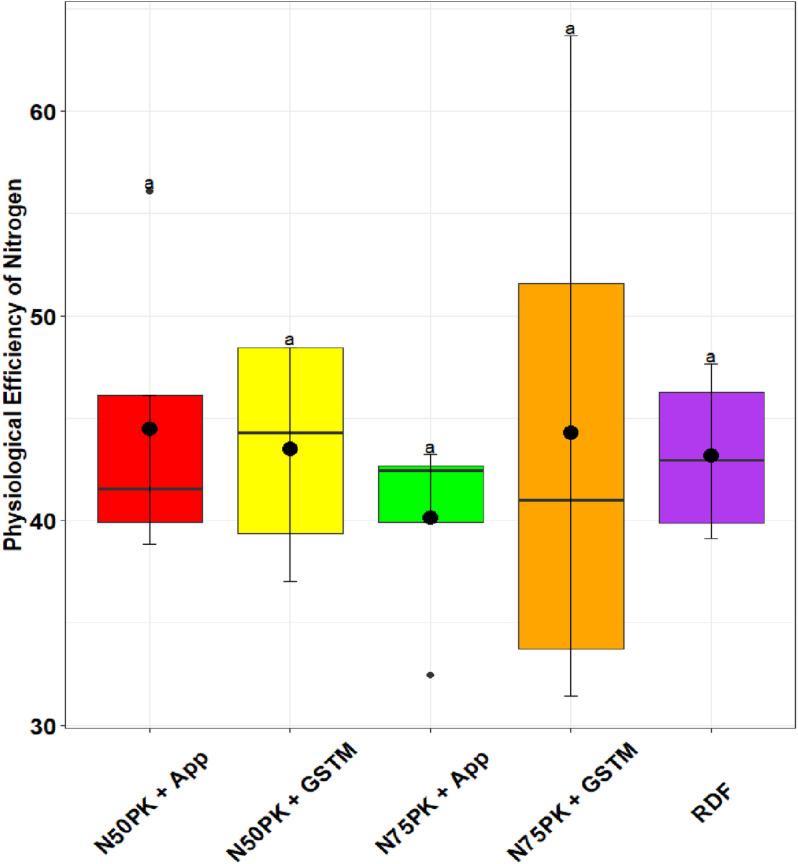
Effect of nitrogen management practices on the Physiological efficiency of nitrogen in maize. The different lowercase letters correspond to the treatments are significantly different at p <  0.05 according to LSD.

**Fig 6:**
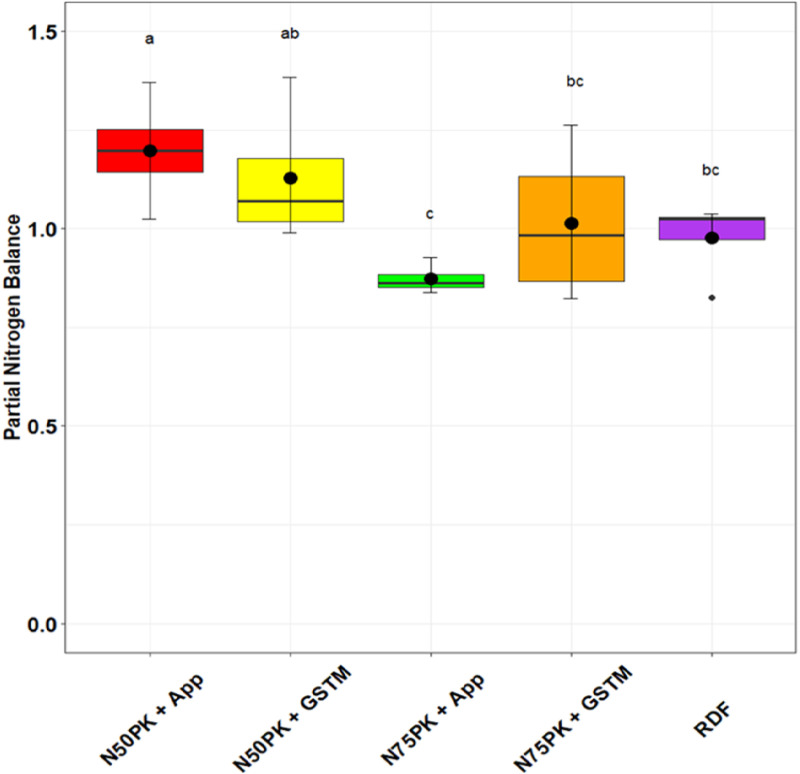
Effect of nitrogen management practices on the Partial Nitrogen Balance in maize. The different lowercase letters correspond to the treatments are significantly different at p <  0.05 according to LSD.

**Fig 7:**
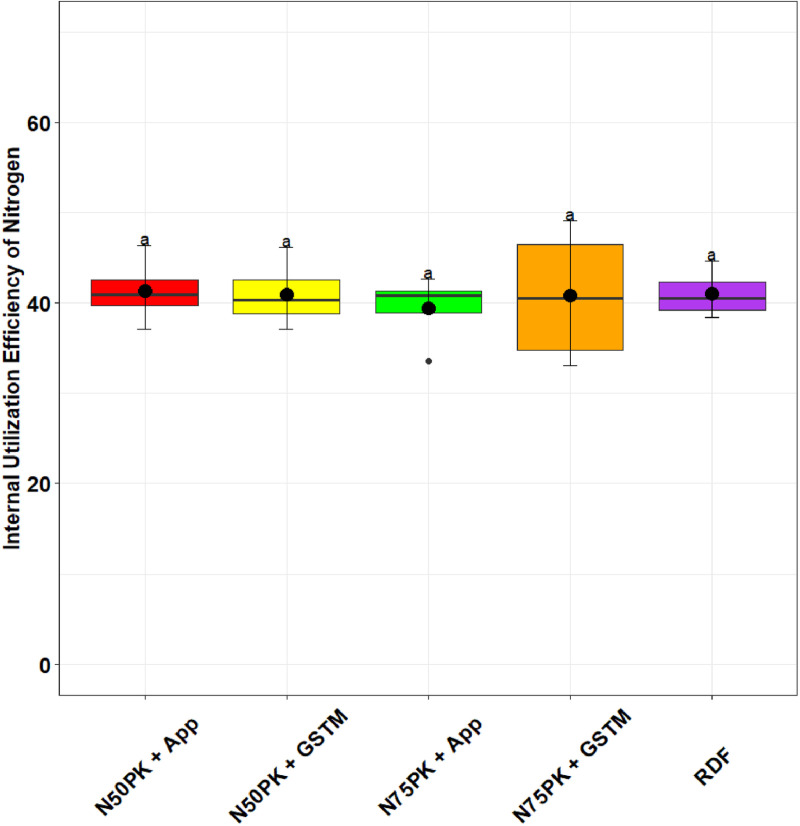
Effect of nitrogen management practices on the Internal Utilization Efficiency of Nitrogen in maize. The different lowercase letters correspond to the treatments are significantly different at p <  0.05 according to LSD.

**Fig 8:**
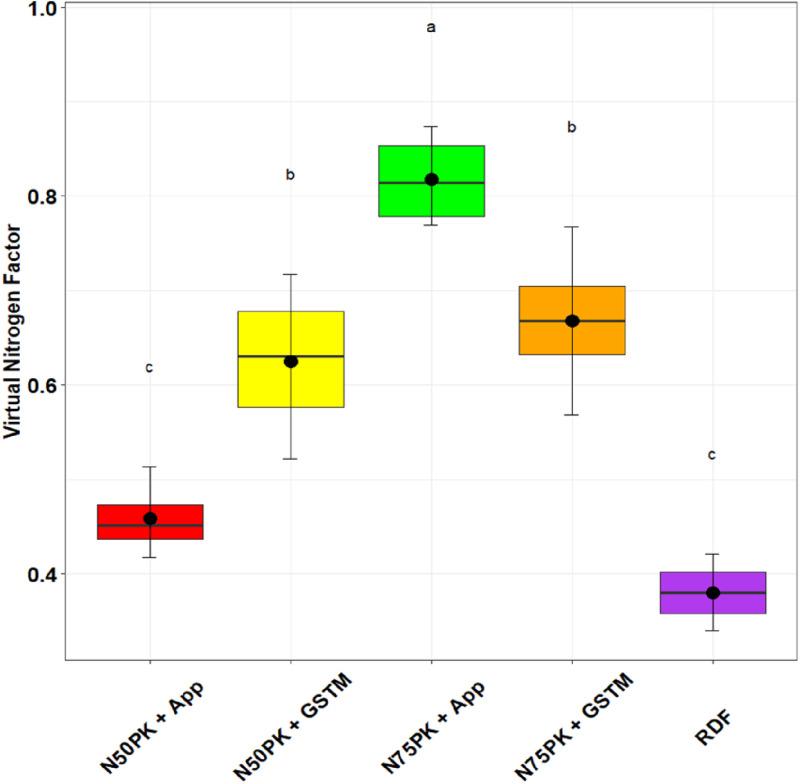
Effect of nitrogen management practices on the Virtual Nitrogen Factor in maize. The different lowercase letters correspond to the treatments are significantly different at p <  0.05 according to LSD.

The N management practices did not influence the PE_N_ significantly. The highest physiological efficiency was in N_50_PK+App (44.5), but was at par with N_75_PK+App (40.15) and other N fertilized treatments (Fig 5). Except N_75_PK+App and RDF, all the N fertilized plots had PNB greater than but close to 1 (Fig 6). The PNB in N_50_PK+App (1.19) had no significant difference with N_50_PK+GS^TM^ (1.12), while the lowest PNB was in N_75_PK+App (0.87). All treatments had statistically comparable IUE_N_ (Fig 7), though it was highest in N_50_PK+App. N_75_PK+App had the lowest IUE_N_. N_50_PK+App had the lowest VNF of 0.45 (Fig 8) which was comparable with RDF (0.38). This was followed by N_50_PK+GS^TM^ and N_75_PK+GS^TM^ having VNF of 0.63 and 0.67 respectively. The N_75_PK+App had the highest VNF, which was 2.16 and 1.8 times the value under RDF and N_50_PK+App respectively.

### 3.6. Saving of cost over RDF, greenhouse gas intensity of nitrous oxide and eco-efficiency index

**Fig 9:**
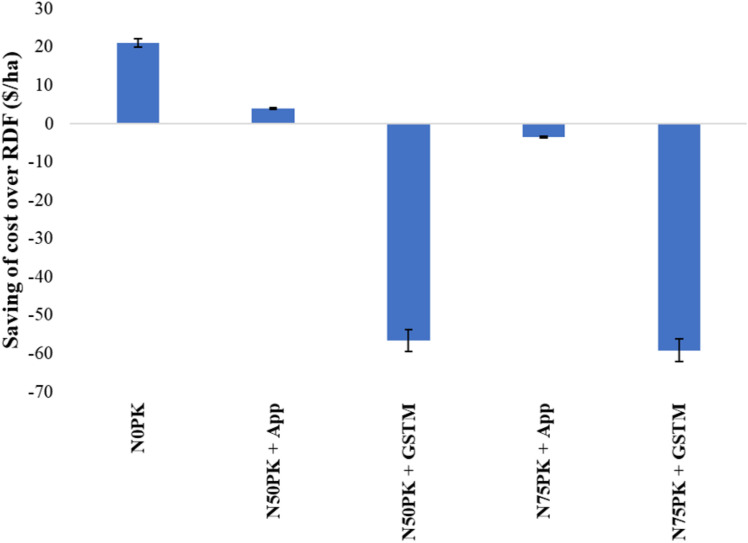
Effect of different nitrogen management practices on the saving of cost over RDF ($/ha). Vertical bars represent standard error (SE) within each treatment. For treatment details, please refer Table 2.

**Fig 10:**
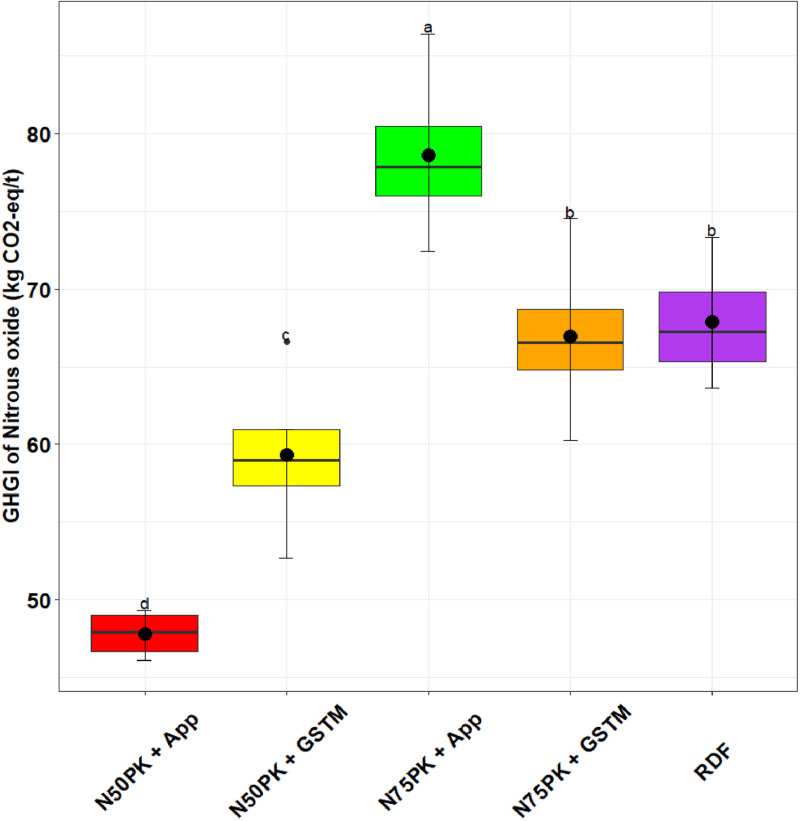
Effect of nitrogen management practices on the GHGI of Nitrous oxide (kg CO_2_-eq/t). The different lowercase letters correspond to the treatments are significantly different at p <  0.05 according to LSD.

Among the N fertilized plots, N_50_PK+App witnessed the maximum saving of 4 US $/ha over RDF. All other treatments except the control had increased CoC over RDF (Fig 9). The GHGI of N_2_O was lowest in N_50_PK+App (Fig 10), which was 29.5% lower than RDF. N_50_PK+App was followed by N_50_PK+GS^TM^ (59.3 kg CO_2_-eq/t). N_75_PK+App had the highest GHGI of N_2_O, being significantly (15.9%) higher than RDF, followed by N_75_PK+GS^TM^ (66.9 kg CO_2_-eq/t).

**Fig 11:**
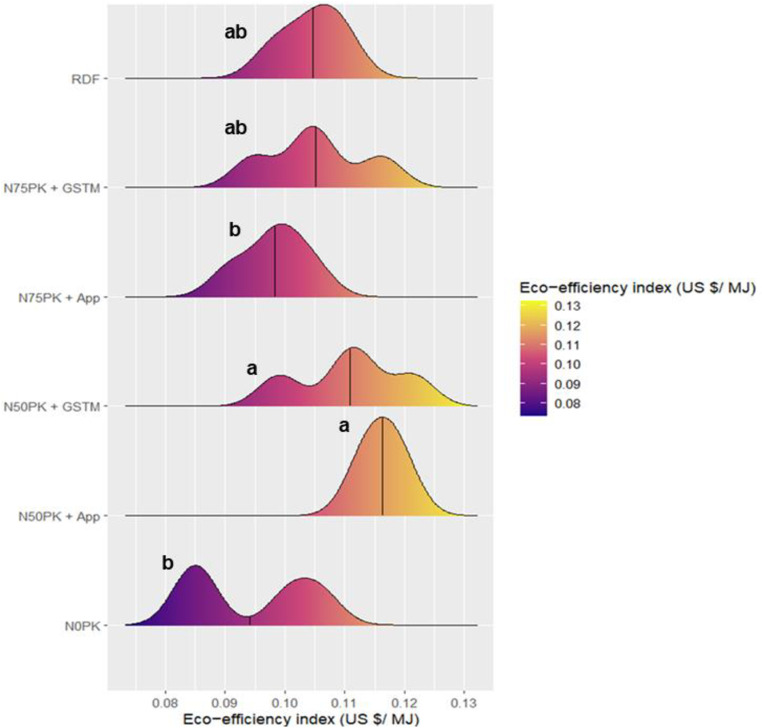
Effect of nitrogen management practices on the Eco-efficiency index (US $/MJ) in maize. The black line within the plot represents the mean. The different lowercase letters correspond to the treatments are significantly different at p <  0.05 according to LSD.

**Fig 12:**
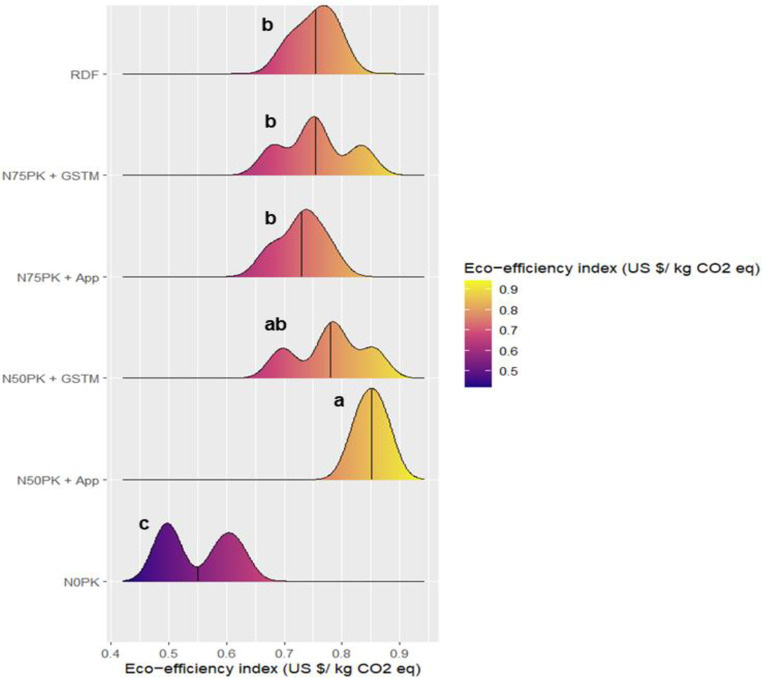
Effect of nitrogen management practices on the Eco-efficiency index (US $/kg CO_2_ eq) in maize. The black line within the plot represents the mean. The different lowercase letters correspond to the treatments are significantly different at p <  0.05 according to LSD.

N_50_PK+App had the highest energy based eco-efficiency index of 0.116 US $/ MJ, which was 11.6% higher than RDF was statistically at par with RDF, N_50_PK+GS^TM^ and N_50_PK+App (Fig 11). The control plot had the lowest energy based eco-efficiency index of 0.094 US $/ MJ, which had no significant difference with N_75_PK+App (0.098 US $/ MJ). Similar was the trend for GHG emission based eco-efficiency index as well, where the highest value was in N_50_PK+App (0.85 US $/ kg CO2 eq) which had no significant difference with N_50_PK+GS^TM^ (0.78 US $/ kg CO2 eq) (Fig 12). This was followed by N_75_PK+GS^TM^ (0.76 US $/ kg CO2 eq) and RDF (0.75 US $/ kg CO2 eq), progressively decreasing in N_75_PK+App (0.72 US $/ kg CO2 eq), with the lowest value in control (0.55 US $/ kg CO2 eq).

## 4. Discussion

### 4.1. Crop growth parameters

The rate of N application is highly correlated with the growth parameters of a crop [36, 37, 38, 39]. In our study, the highest cumulative dose of N was applied in N_75_PK+App treatment, which improved the maize plant height, LAI as well the dry matter accumulation. Similar to our findings, Hammad et al. [40], reported a higher maize plant height and dry matter accumulation with a higher dose of N application in maize. The addition of N boosted cell counts and leaf volume, sped up chlorophyll production, and enhanced plant biomass in the early stages of crop development [41]. Moreover, Amanullah et al. [42], also observed a positive correlation between LAI and fertilizer N dose in maize. Similar results have been reported by Amanullah and Shah [43], Ning et al. [44], Zhang et al. [45], Patra et al. [39], Deng et al. [46], who also found enhanced growth, LAI, photosynthetic rate and biomass accumulation in maize with an increment in the N fertilizer application rate.

While the CGR at (0-30) DAS was maximum in N_75_PK+App, but the RGR followed an opposite trend where the N_50_PK+App had the highest RGR and it was least in N_75_PK+App. This might be because, a small plant that is growing relatively quickly can exhibit high RGR at its early stages, as RGR depends on the initial biomass of the plant and the relative increase in biomass is significant [47, 48]. On the other hand, CGR is more influenced by total biomass increase per unit area and time, which might be low resulting in a lower CGR [49, 50].

### 4.2. Shelling percentage and harvest index

The highest shelling percent in maize was achieved in N_50_PK+App, which was statistically at par with the other N fertilized plots and the no N control. The shelling percentage in maize may not improve with N application compared to control plot because shelling percentage is primarily influenced by the ratio of grain to cob. N application often boosts overall plant growth, including both grain and cob development, but the relative increase in grain weight might be proportionate to the cob, leading to little or no change in the shelling percentage. The lowest harvest index was in the control plot, followed by N_75_PK+App treatment, which had no statistically significant difference with the other precision N based treatments and RDF. Similar to our finding, Zhang et al. [45], found at par harvest index across the different N application treatments. Moreover, field experiment on maize in Ethiopia showed that increasing N rates consistently boosted the harvest index until reaching the maximum application of 115 kg N/ha [51].

### 4.3. Nitrogen content in plant at 40 and 50 DAS, and in grain and stover at harvest

At Both 40 and 50 DAS the N content in maize plant as well as the N content in maize grain and stover at harvest was highest in the treatments applied with 75 kg N as basal, while the lowest values were in the treatments applied with 50 kg N as basal, though all the treatments were statistically at par. There was no significant difference amongst the N fertilized plots with that of the no N control due to a dilution effect: as N fertilizer rates increase, so does dry matter production, which dilutes the N concentration in the plant [52, 53]. With lower dry matter production, the N content is relatively higher per unit of dry matter [54, 55]. Therefore, even though N application increases both dry matter and N uptake, the N concentration per 100 kg of dry matter remains similar between the control and the N-fertilized plots [56]. Mondal et al. [57] also noted that the N content in maize and stover was comparable, but the stover yield varied significantly across different fertilizer N treatments. The varying N application rates of 0, 120 and 150 kg N/ ha did not lead to statistically significant differences in the N content of the grain between treatments in a subsurface drip fertigated conservation agriculture system [39].

### 4.4. Nitrogen use efficiency

In our study, all the treatments were at par with each other in terms of their physiological efficiency of N. Physiological efficiency is the efficiency with which absorbed N is converted into biomass or grain [58]. This metric is determined by the plant’s inherent capacity to use the absorbed N for growth and development [24]. Since physiological efficiency is a function of the plant’s internal metabolism and indicates the proportional increase in yield with increase in N plant uptake, it remained stable across different fertilizer treatments [59, 60].

Typically, a PNB value greater than 1 indicates that more nutrients are being extracted from the soil through crop harvesting than are being replenished by fertilizers or manure [61, 62]. This imbalance can result in the depletion of soil nutrients, a process known as “soil mining.” In our study, PNB was greater than but close to 1 in most of the N fertilized plots except N_75_PK+App and RDF. This can be because PNB is a partial balance and does not consider the indigenous soil N supply [63], thereby indicating that a PNB greater than 1 might not always lead to soil N mining. The lowest PNB was in N_75_PK+App, which is lower than 1, indicates a significant amount of N loss, due to application of excess amount of N than the crop requirement, further leading to environmental deterioration [64].

The IUE_N_ is relatively stable across different N fertilized treatments. Since IUE_N_ is the ratio of grain yield to total N uptake, it implies that both the grain yield and N uptake increase or decrease proportionally [25]. As a result, even though there may be variations in grain yield and N uptake between treatments, the efficiency itself remains consistent, which is why no statistically significant difference is observed across treatments. Similar to our result, Qiu et al. [26], in field experiments in three villages of Jilin, China, observed that IUE_N_ showed little variation when N was applied at rates of 140, 210, and 280 kg/ha. The highest IUE_N_ in N_50_PK+App, with least value in N_75_PK+App, is due to the moderately high grain yield with a low total N uptake [65] in N_50_PK+App, and highest grain yield and total N uptake in N_75_PK+App.

The N_75_PK+App had the highest VNF, which was mainly attributed to its highest N loss, significantly higher than other N fertilized plots. The lowest VNF in RDF, which was at par with N_50_PK+App, can be explained by a high grain N uptake, with low N loss.

### 4.5. Saving of cost over RDF, greenhouse gas intensity of nitrous oxide and eco-efficiency index

Saving of cost over RDF was highest in control plot followed by N_50_PK+App, and all other treatments had increased cost over RDF. This was because of no nitrogenous fertilizer application and the lowest dose of fertilizer N application in control and N_50_PK+App respectively. The remaining treatments had higher CoC compared to RDF, because of the highest dose of N fertilizer application in N_75_PK+App, and consideration of the cost of the GS^TM^ during CoC computation in the GS^TM^ based treatments.

The GHGI of N₂O was highest in the N_75_PK+App treatment, while the lowest value was recorded in the N_50_PK+App treatment. Applying a higher dose of N enhances the soil N content. This surplus N is more likely to undergo various microbial activities, like nitrification and denitrification. During these processes, microbes convert excess N into N₂O, a potent GHG [66]. Halli et al. [34], delineated that the use of nitrogenous fertilizer had a direct response to N₂O emission rate. Specifically, applying N in both basal and split doses at 100 and 120 kg/ha, respectively, led to higher N₂O emissions of 2.14 kg/ha, compared to 1.08 kg/ha in untreated plots. In the maize-wheat cropping system, N fertilizer was the chief causal factor to direct N₂O emissions, making up 65.9% of the cumulative emissions [67]. Chai et al. [68] determined that the N fertilizer use in wheat and maize resulted in 35.82 Gg/year and 69.44 Gg/year N₂O emission respectively in China. A study conducted over two years at five farms in Michigan, USA, investigated maize with six rates of N ranging between 0 to 225 kg/ha per season. The research consistently found that N₂O emissions increased exponentially with higher N rates at each site, regardless of the year [69, 70]. Moreover, a global meta-data analysis, comprising of 78 studies spanning 233 sites, found that the N₂O emissions rose exponentially once the N rate surpassed the crop requirement [71, 72].

N_50_ based treatments (N_50_PK+App and N_50_PK+GS^TM^) showed the highest energy based as well as GHG emission based eco-efficiency index, with lowest value in N_75_PK+App. This trend was opposite to the N dose applied to the crop. In the overall energy expenditure in agricultural systems, fertilizer, being the most energy-demanding input [73, 74], contributed the largest share due to its non-renewable nature [32], and accordingly led to the lowest energy based eco-efficiency index value in N_75_PK+App, applied with the highest N dose. Analogous to this result, the increased application of N fertilizers also has a higher carbon footprint due to the energy-intensive character of fertilizer manufacture, transport, application [33], along with nitrous oxide emission from soil, which attributed to the lowest GHG emission based eco-efficiency index in N_75_PK+App. A study in Southern Italy found that higher yields and economic returns don’t always offset the increased resource use, reducing eco-efficiency [75]. Low-resource farming, especially with minimal N use, proved more eco-efficient than intensive methods [76].

## 5. Conclusion

The Pusa N Doctor app, utilizing a DGCI-based algorithm, effectively optimized nitrogen management in maize by improving N use efficiency, reducing GHG emissions, and enhancing eco-efficiency. The app demonstrated superior results in comparison to traditional tools like GreenSeeker™ particularly in terms of cost-effectiveness and precision in N management. By recommending a basal N application of 50 kg with split applications, it ensures sustained maize growth, efficient N use, and a reduction in environmental impacts, making it a promising alternative to existing nitrogen management tools. Furthermore, the implementation of such innovative digital tools has the potential to revolutionize precision agriculture by providing scalable and farmer-friendly solutions. Future research could focus on refining the app for diverse cropping systems and agro-climatic conditions, while policy integration could enhance adoption rates, ultimately contributing to sustainable agriculture and climate change mitigation on a global scale.

## Abbreviations

SMW: standard meteorological week
Tmax: maximum temperature
Tmin: minimum temperature
RH (E): Relative humidity evening
RH (M): Relative humidity morning
